# Editorial: Inflammatory Cells in the Sick Heart and Adipose Tissue: Novel Targets for Old and New Drugs

**DOI:** 10.3389/fphys.2020.612228

**Published:** 2021-02-02

**Authors:** Laura Raimondi, Angelo Parini

**Affiliations:** ^1^University of Florence, Florence, Italy; ^2^Institute of Cardiovascular and Metabolic Diseases, Inserm U1048, Tolouse, France; ^3^Université Toulouse III Paul Sabatier, Toulouse, France

**Keywords:** inflammation, epicardial adipose tissue, adipokines, atrial fibrillation, serine proteases

The heart is among the target organs of adipose tissue-derived paracrine/endocrine signals. These include adipokines and cytokines but also inflammatory and hypertrophic signals such as serine proteases. Consistently, changes in the levels/quality of adipose tissue proteomic profile, as it occurs in obesity, impact on heart health and take part in the onset and exacerbation of cardiac diseases, including atherogenic-based diseases and arrhythmias, which all have a common pathogenic ground, i.e., inflammation, and a certain degree of insulin resistance. A particular type of adipose tissue is the epicardial adipose tissue (EAT), an ectopic deposition of visceral fat that shares microcirculation (i.e., the coronary arteries) with the myocardium, and presents its own innervation consisting of adrenergic and cholinergic fibers.

EAT is a beige/white adipose tissue that maintains the plasticity of the fat cell and whose physiological role is not fully elucidated but that includes mechanical, trophic, and thermogenic functions. As a visceral fat pad, EAT produces multiple pro- and anti-inflammatory adipokines and cytokines, it secretes a large amount of fatty acids and expresses signaling pathways for serine proteases, including coagulation factors. Evidence indicates that an increase of EAT mass and that the extent of tissue inflammation with dense macrophage infiltration, vascular inflammation, and over-activation of the adrenergic system, is associated with the presence of coronary artery diseases (CAD), heart failure (HF), and atrial fibrillation (AF). The process by which tissue becomes sick, first in the heart, in vessels or the EAT, and the identification of the predictive markers of sickness, as well as the impact of EAT thickness on cardiac disease prognosis, have become subjects of interest in recent clinical research, and the three research papers and the review collected in this Research Topic explore these issues further.

In the context of HF, Parisi, Petraglia et al. and Agra-Bermejo et al. studied the contribution of EAT mass and EAT-derived inflammatory mediators in predicting HF prognosis. in a cohort of patients with HF with compromised LV function. Parisi, Conte et al. reported that EAT thickness predicted clinical outcomes including episodes of paroxysmal AF, HF-related deaths, and the hospitalization rate for HF. This study proposes the measure of EAT mass as a clinical indicator of HF progression and an active role of EAT in the formation of the AF substrate. The same authors compared the levels and possibly the biological effectiveness of the pro-atherogenic IL-1β in EAT biopsies and the plasma of patients with chronic, stable CAD, in CAD patients undergoing coronary artery bypass grafting in non-CAD patients (controls). Interestingly, the overall plasma and EAT IL-1β levels/ effectiveness were found to be higher in CAD vs. control patients. In the CAD groups, patients undergoing bypass had higher IL-1β levels than those with stable CAD. Interestingly, plasma and EAT IL-1β levels positively correlated in the overall CAD population with adipocytes mainly participating in IL-1β production. Such data indicate that the plasma levels of the pro-inflammatory IL-1β mirror its concentration in the EAT, and suggests that they could be considered a surrogate index of inflammation and the role of EAT in the progression of heart sickness. This interesting conclusion needs to be corroborated by further and larger clinical studies.

Agra-Bermejo et al. investigated the effect of neurohumoral dysregulation, i.e., adrenergic over-activation, on the release of fat-derived proteins possibly predictive of AF in HF patients. They studied the secretome of EAT biopsies from HF patients with AF or patients with HF with sinus rhythm undergoing bypass surgery. This latter population was then followed for 5 years and monitored for AF occurrence. The authors noted that the stimulation with isoproterenol induced the secretion of numerous proteins of which, 17 were present both in the secretome of EAT from patients with stable or paroxysmal atrial fibrillation. Among which, the CD5L, a macrophage apoptosis inhibitor and an immune competent mediator involved in inflammation and extracellular matrix protein formation/deposition CD5L levels, were found to be higher in the secretome of EAT from AF vs. not-AF patients, an increase which reached a level of significance in the EAT taken from male AF patients. This result indicates that among the signals which mediate the pro-fibrotic effect of adrenergic over-activation, CD5L secretion may represent an early and gender-specific biomarker of EAT inflammation and then of AF occurrence. Should CD5L predictiveness of AF in male subjects be confirmed by larger future studies, a gender-specific contribution in the formation of AF substrate could be postulated, which could facilitate the investigation of specific pharmacological tools. In this respect, Coppini et al. analyzed a series of possible therapeutic strategies to reduce cardiac fibrosis, i.e., AF substrates, focusing on the pharmacological inhibitors of mastocyte and macrophage-derived serine proteases, an enzyme involved in multiple processes including activation/inactivation of biological peptides endowed with unique cell signals. The authors examined the pathways activated by these enzymes and their involvement in cardiac fibrosis and highlighted the role of pharmacological enzyme inhibitors, among which direct anticoagulants, oral and parenteral, DPPIV inhibitors, may play a role in reducing AF substrate formation. The authors' conclusion reveals the interesting potential of these drugs as disease-modifying agents.

Obesity is characterized by the hypertrophy of existing adipocytes and the recruitment of novel adipose cells. These aberrant pathways are associated with tissue immune-inflammatory cell infiltration. Visceral obesity represents a risk factor for ectopic fat deposition, i.e., lipotoxicity. Effective therapies for obesity are based on the amelioration of insulin resistance or stimulation of lipid degradation. reported Qiao et al. on the novel pharmacological effect of hydroxychloroquine, a potent immunosuppressive agent that has recently received attention as a possible treatment for Covid-19 infection. The authors reported that hydroxychloroquine was effective in reducing insulin resistance, obesity, and liver steatosis by activating the peroxisome activated receptor gamma (PPARg) in mice fed with a high-fat diet. This evidence adds knowledge on hydroxychloroquine pharmacological profile and suggests drug effectiveness in ameliorating fat inflammation independently of the presence of clinically relevant autoimmunity. However, it is known that the long-term effects of PPARg agonists include activation of adipocyte hypertrophy and then of pro-inflammatory pathways, prompting the generation of atrial fibrillation substrates.

The original articles, review, and research cited in this Research Topic present novel possible markers of fat sickness and their possible prognostic role in the progression of cardiac disease.

## Author Contributions

LR and AP contributed to write the paper. All authors listed have made a substantial, direct and intellectual contribution to the work, and approved it for publication.

## Conflict of Interest

The authors declare that the research was conducted in the absence of any commercial or financial relationships that could be construed as a potential conflict of interest.

